# Thinking About the Nerve Impulse: The Prospects for the Development of a Comprehensive Account of Nerve Impulse Propagation

**DOI:** 10.3389/fncel.2019.00208

**Published:** 2019-05-15

**Authors:** Linda Holland, Henk W. de Regt, Benjamin Drukarch

**Affiliations:** ^1^Amsterdam Neuroscience, Department of Anatomy and Neurosciences, Vrije Universiteit Amsterdam, Amsterdam UMC, Amsterdam, Netherlands; ^2^Department of Philosophy, Faculty of Humanities, Vrije Universiteit Amsterdam, Amsterdam, Netherlands

**Keywords:** nerve impulse propagation, action potential, Hodgkin-Huxley model, soliton model, comprehensive modeling, complete representation, model as tool, comprehensive framework

## Abstract

Currently, a scientific debate is ongoing about modeling nerve impulse propagation. One of the models discussed is the celebrated Hodgkin-Huxley model of the action potential, which is central to the electricity-centered conception of the nerve impulse that dominates contemporary neuroscience. However, this model cannot represent the nerve impulse completely, since it does not take into account non-electrical manifestations of the nerve impulse for which there is ample experimental evidence. As a result, alternative models of nerve impulse propagation have been proposed in contemporary (neuro)scientific literature. One of these models is the Heimburg-Jackson model, according to which the nerve impulse is an electromechanical density pulse in the neural membrane. This model is usually contrasted with the Hodgkin-Huxley model and is supposed to potentially be able to replace the latter. However, instead of contrasting these models of nerve impulse propagation, another approach integrates these models in a general unifying model. This general unifying model, the Engelbrecht model, is developed to unify all relevant manifestations of the nerve impulse and their interaction(s). Here, we want to contribute to the debate about modeling nerve impulse propagation by conceptually analyzing the Engelbrecht model. Combining the results of this conceptual analysis with insights from philosophy of science, we make recommendations for the study of nerve impulse propagation. The first conclusion of this analysis is that attempts to develop models that represent the nerve impulse accurately and completely appear unfeasible. Instead, models are and should be used as tools to study nerve impulse propagation for varying purposes, representing the nerve impulse accurately and completely enough to achieve the specified goals. The second conclusion is that integrating distinct models into a general unifying model that provides a consistent picture of nerve impulse propagation is impossible due to the distinct purposes for which they are developed and the conflicting assumptions these purposes often require. Instead of explaining nerve impulse propagation with a single general unifying model, it appears advisable to explain this complex phenomenon using a ‘mosaic’ framework of models in which each model provides a partial explanation of nerve impulse propagation.

## Introduction

In a celebrated paper, Hodgkin and Huxley (1952a) presented a model with which they provided a quantitative description of the electrical events underlying the generation and propagation of a nerve impulse. This model is still vitally important in the neurosciences and is the foundation for a broad area of neuroscientific research (Catterall et al., 2012). The ‘Hodgkin-Huxley’ (HH) model was the result of a long period of electricity-centered study in electrophysiology that had started in 1791 with the work of Galvani (Piccolino, 1998; Drukarch et al., 2018). In line with its history, the model considers the nerve impulse as a purely electrical pulse or ‘action potential’. It describes the action potential as the result of ion fluxes across the neural membrane due to an ion-specific change in membrane permeability upon an alteration in the membrane potential (Hodgkin and Huxley, 1952a).

The behavior of the HH model nerve is in good agreement with several electrical properties of the (propagated) nerve impulse in experiments. However, the model cannot account for non-electrical manifestations of the nerve impulse for which there is ample experimental evidence. Changes that are found to occur in association with nerve impulse propagation include, but are not restricted to, mechanical and thermal changes (reviewed in Drukarch et al., 2018). These changes could have functional importance in nerve impulse propagation (Costa et al., 2018). However, whether they do, and, if so, how they are related to the electrical aspect of the nerve impulse, is still a matter of debate (Mueller and Tyler, 2014; El Hady and Machta, 2015).

Notwithstanding the remaining uncertainties, opinions have been voiced that the available experimental evidence asks for a more comprehensive consideration of the nerve impulse that accounts for electrical as well as non-electrical aspects of this phenomenon, rather than representing it as a solely electrical event (Andersen et al., 2009; Mueller and Tyler, 2014). Consequently, in neuroscientific literature, alternative models have appeared that attempt to take into account electrical and non-electrical changes associated with nerve impulse propagation (Heimburg and Jackson, 2005; Rvachev, 2010; El Hady and Machta, 2015). In one of the proposed models, the ‘Heimburg-Jackson’ model, the nerve impulse is considered to be a propagating density pulse in the neural membrane. In this model, the focus is shifted from membrane proteins, i.e., ion channels (which play an important role in nerve impulse generation and propagation according to the view that evolved after introduction of the HH model), to membrane lipids. It is usually contrasted with the HH model (Heimburg and Jackson, 2006; Andersen et al., 2009; Appali et al., 2012). Moreover, the Heimburg-Jackson model is designated as a potentially revolutionary model that challenges neuroscientific dogmas about nerve impulse propagation (Fox, 2018, reprinted in the Special Editions Volume 27 of *Scientific American* entitled ‘Revolutions in Science’; Meissner, 2018). Currently, there is an active debate whether the Heimburg-Jackson model (which is supported by experimental measurements in (artificial lipid) membranes (Heimburg and Jackson, 2005; Wang et al., 2018) and some neuronal models (Gonzalez-Perez et al., 2014, 2016; Wang et al., 2018) but is still largely theoretical in nature) can replace the HH model, and several tests to decide between these models have been proposed (Meissner, 2018). However, although more extensive experimental validation is important, this should not be the only perspective from which alternative models are evaluated. It should be complemented with a conceptual analysis that investigates distinct models of nerve impulse propagation and discusses their role in studying this complex phenomenon. Such an analysis is needed since experimental data can always be interpreted in different ways, and additional arguments are needed to decide which interpretation of these data is superior.

In addition, instead of contrasting these different approaches to nerve impulse propagation, it is also argued in current (neuro)scientific literature that views focusing on membrane proteins and membrane lipids should be integrated in a general unifying model. Such a general unifying model is developed to incorporate, integrate and explain all relevant aspects of the nerve impulse by unifying different manifestations of the nerve impulse and the interaction(s) between them (Mueller and Tyler, 2014; Engelbrecht et al., 2018b). An important argument for developing such a general unifying model is to obtain insights in nerve impulse propagation that cannot be acquired using models that focus on only one or a few aspects of the nerve impulse without studying the interactions between them. In Mueller and Tyler’s words: “To advance our understanding of how nervous systems operate it is important to develop comprehensive models where electrical, chemical, and mechanical energies are not compartmentalized from one another, but rather cooperate in a synergistic manner to regulate neuronal excitability and signaling. By starting to consider the interplay between electrical, chemical, and mechanical energy, new paradigms for understanding and studying the biophysics of neural systems will advance our comprehension of brain function” (Mueller and Tyler, 2014, p. 3).

At first sight, developing a general unifying model seems to be a promising approach for building a comprehensive framework of nerve impulse propagation. However, this proposal also raises several questions. First, is it feasible to actually construct such a model or is this mission too ambitious to accomplish? Second, if it is at least in theory possible to construct such a model, what should the model comprise? And, third, how should we tackle its construction?

In this article, we will conceptually analyze a recently introduced general unifying model, developed by Engelbrecht et al. (2016, 2018a,b). This article builds on a previous article by us (Drukarch et al., 2018) in which we discuss the HH model and alternative models of nerve impulse propagation from a historico-scientific perspective covering the (neuro)scientific literature on the phenomenon of nerve impulse propagation. Here, we follow up on this and discuss some models of nerve impulse propagation from a conceptual point of view in order to conceptually analyze the feasibility of the idea of developing a general unifying model or comprehensive model of nerve impulse propagation, using a recently introduced general unifying model of this phenomenon as an example. Combining the results of our conceptual analysis with recent insights from philosophy of science, we will make some recommendations for the study of nerve impulse propagation. More specifically, we will evaluate in the section “The Engelbrecht Model: An Attempt at a General Unifying Model” whether the ‘Engelbrecht’ model provides a complete and accurate representation of the propagating nerve impulse. Before elaborating on the Engelbrecht model, however, we will examine in the section “The Hodgkin-Huxley Model” whether the standard model of the nerve impulse, the HH model, represents the nerve impulse accurately and completely. In the section “Recommendations for (Future) Approaches to Studying Nerve Impulse Propagation”, we use our analysis of the Engelbrecht model and insights from philosophy of science to formulate recommendations (1) with regard to the role of models in studying nerve impulse propagation and (2) for constructing a comprehensive framework of nerve impulse propagation. Finally, in the concluding section, a possible role for a general unifying model in this comprehensive framework will be discussed. Although a very important issue, we would like to emphasize that the current study is not aimed to discuss or predict the consequences of modeling nerve impulse propagation for different types of nerve fibers using the models referred to here.

## Models as Complete and Accurate Representations of Nerve Impulse Propagation?

### The Hodgkin-Huxley Model

The HH model is often considered to provide a complete and accurate representation of the (propagating) nerve impulse, which according to this model is a purely electrical pulse. In other words, the HH model is usually taken to reflect or mirror the biologically ‘real’ nerve impulse. In this section, we will examine whether the HH model indeed provides such an accurate and complete representation of the nerve impulse. In this discussion, ‘accurate’ is defined as (nearly) “free from error especially as the result of care” and ‘complete’ as “having all necessary parts, elements, or steps” (which is in accordance with the definition of these terms in the online Merriam-Webster dictionary in 2018). We discuss the HH model here, first of all, because it is a vitally important model in the neurosciences (e.g., Catterall et al., 2012). It is the result of a long and impressive research tradition in the neurosciences (for an elaborate review, see Drukarch et al., 2018) and is often confirmed in subsequent neuroscientific studies (e.g., Tasaki and Hagiwara, 1957; Naharashi et al., 1964). Moreover, it has now been accepted as an educational textbook-model of action potential generation and propagation (e.g., Purves et al., 2012). Secondly, because it is a well-known model among neuroscientists, which allows us to illustrate the meaning of the concepts ‘accurate’ and ‘complete’. And, finally, because it embodies the received view to which proposed alternative models of nerve impulse propagation are and have to be related.

The propagating nerve impulse is a phenomenon that cannot be observed directly. Therefore, Hodgkin and Huxley devised experiments to obtain information about this phenomenon. In these experiments they used the voltage clamp technique. With this technique, the membrane potential of an isolated nerve fiber can be changed suddenly, after which it is held constant (clamped) using an electrical feedback circuit. The current that must be injected in the nerve fiber to keep the membrane potential constant is assumed to be similar to the current that flows through the neural membrane (Hodgkin et al., 1952).

**Figure 1:**
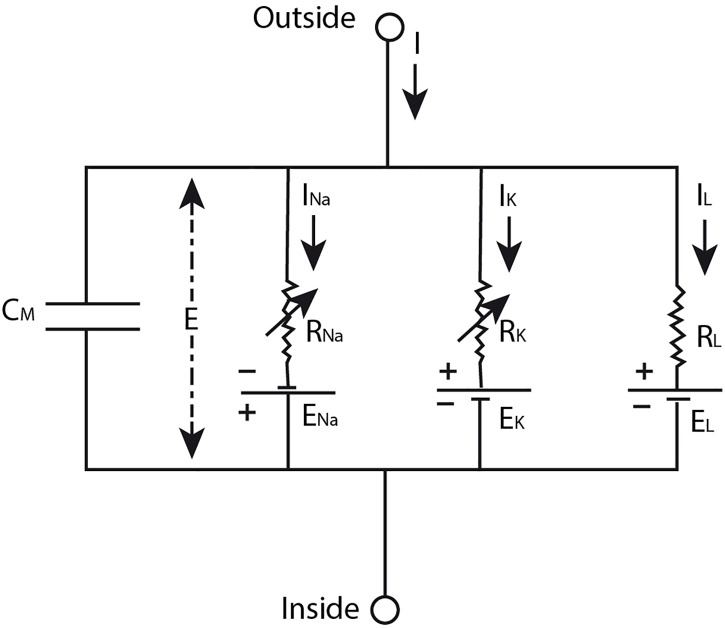
The neural membrane modeled as an electrical circuit. The lipid bilayer membrane is conceptualized as a capacitor (*C*_M_), the ion-specific permeability of the membrane is modeled by the resistors (*R*_ion_), and the electrochemical gradient across the membrane is represented by the batteries (*E*_ion_). In this model, *E* designates the membrane potential, *I* the total current through the membrane, and *I*_ion_ the ionic currents of sodium ions (Na), potassium ions (K), and a leakage (L) current of other ions. Source figure (redrawn): Hodgkin and Huxley. A quantitative description of membrane current and its application to conduction and excitation in nerve. *The Journal of Physiology*. John Wiley & Sons, Inc.

On the basis of data that Hodgkin and Huxley gathered in their experiments (Hodgkin and Huxley, 1952b,c,d; Hodgkin et al., 1952) they developed a model (Hodgkin and Huxley, 1952a) in which the nerve impulse is described as the result of “a capacity current which involves a change in ion density at the outer and inner surfaces of the membrane, and an ionic current which depends on the movement of charged particles through the membrane” (Hodgkin et al., 1952, p. 426) upon depolarization of the membrane. The ionic current can be further divided in currents of sodium and potassium ions and a leakage current of other ions. The sodium and potassium ions travel down their electrochemical gradient across the membrane that is selectively permeable for them during different phases of the nerve impulse (Hodgkin and Huxley, 1952a). More specifically, Hodgkin and Huxley modeled the neural membrane as an electrical circuit (Figure 1), consisting of a capacitor representing the lipid bilayer, resistors conceptualizing the ion-specific membrane permeability, and batteries modeling the concentration gradient across the membrane that drives the flow of ionic current through the membrane. The mathematical equation that can be derived from this electrical circuit describes the total current density through the membrane quantitatively (see Equation 1). To model the propagating nerve impulse, this equation had to be extended in order to take into account the current flow along the nerve fiber as well (Hodgkin and Huxley, 1952a). However, we will not discuss this extended equation here to avoid unnecessary complexity.

**EQUATION 1. Hodgkin-Huxley equation describing the total membrane current.**

$$I\;=\;\left[1\right]\;C_{M}\;\frac{dV}{dt}\;+\;\left[2\right]\;g_{K}\;\left(V\;-\;V_{K}\right)\;+\;\left[3\right]\;g_{Na}\;\left(V\;-\;V_{Na}\right)\;+\;\left[4\right]\;g_{L}\;\left(V\;-\;V_{L}\right)$$

In this equation, term [1] describes the capacity current, which depends on the membrane capacitance (*C*_M_) and the change in the displacement of the membrane voltage from its resting value over time $\left(\frac{dV}{dt}\right)$, and the other terms describe the total ionic current, which consists of [2] a potassium ion (*K*) current, [3] a sodium ion (*Na*) current and [4] a leakage (*L*) current of other ions. Each ionic current is determined by the ionic permeability of the membrane which is described in terms of an ionic conductance (*g*_ion_, which is the inverse of the electrical resistance) and a driving force that is the result of the difference between the displacement of the membrane potential from its resting value (*V*) and the equilibrium potential for the ions given as a displacement from the resting membrane potential (*V*_ion_) (Hodgkin and Huxley, 1952a).

At the time when Hodgkin and Huxley developed and introduced their model, the “thickness and composition of the excitable membrane” were unknown (Hodgkin and Huxley, 1952a, p. 501). Therefore, they did not know *how* sodium and potassium ions pass the nerve fiber membrane. For this reason, they tried to find equations for the sodium and potassium conductance terms in Equation 1 “which describe the conductances with reasonable accuracy and are sufficiently simple for theoretical calculation of the action potential” by comparing theoretical equations with experimental data (Hodgkin and Huxley, 1952a, p. 506). Equation 1 is thus a simplified version of the equation that Hodgkin and Huxley developed to describe the total current through the membrane. In the complete equation, the terms for the potassium and sodium conductance are given by ion-specific constants and ion-specific dimensionless variables. The changes of these variables over time are described in separate differential equations. The rate constants of these differential equations are in turn given by additional equations (Hodgkin and Huxley, 1952a). For the same reason as mentioned above, these equations will not be discussed here.

Since Hodgkin and Huxley developed phenomenological equations for the sodium and potassium conductance of the membrane, these equations are “an empirical description of the time-course of the changes in permeability to sodium and potassium” (Hodgkin and Huxley, 1952a, p. 541). For this reason, it cannot be concluded that the HH model provides an accurate representation of the nerve impulse, because “[a]n equally satisfactory description of the voltage clamp data could no doubt have been achieved with equations of very different form, which would probably have been equally successful in predicting the electrical behavior of the membrane” (Hodgkin and Huxley, 1952a, p. 541). Although Hodgkin and Huxley limited the possible explanations of the conductance changes of the neural membrane considerably with their experiments and model (e.g., they excluded the possibility that the membrane breaks down in a non-specific manner allowing non-specific ion flow through the membrane), the conductance equations that Hodgkin and Huxley developed do not provide evidence in favor of a certain mechanism of membrane permeability, they could not provide “any certain information about the nature of the molecular events underlying changes in permeability” (Hodgkin and Huxley, 1952a, p. 501).

A few decades later a new technique was developed, the patch clamp (Neher and Sakmann, 1976). With the patch clamp technique, which is a refinement of the voltage clamp technique, the current flowing through small patches of the membrane can be measured. In experiments involving the patch clamp, evidence could be provided that the ion flow through the membrane is localized to ion channels embedded in the membrane (e.g., Sigworth and Neher, 1980). Thus, the HH model could be supplied with a physical interpretation of the ion conductance through the membrane, and in combination with this additional information the nerve impulse could be represented accurately with the HH model^1^.

However, does the HH model also represent the nerve impulse completely? Hodgkin and Huxley stood in a research tradition that had started in the 18th century, the electrophysiological research tradition. In electrophysiology, the electrical nature of the nerve impulse was (and is) generally accepted and intensively studied (for a historical overview, see Clower, 1998; Piccolino, 1998; Drukarch et al., 2018). Electrophysiologists investigated the action potential in increasing detail, providing insights in the form of the action potential, the velocity of its conduction, the importance of the neural membrane for the generation and propagation of action potentials, and the selective nature of membrane permeability. Hodgkin and Huxley (1952a,b,c,d) especially added insights to the last item on this list, and thereby contributed to an explanation of the time-course of the membrane voltage during the action potential.

Due to this long tradition of electrophysiological research in which electricity was the main focus for hypothesizing about, experimenting on and modeling of the nerve impulse, we understand the action potential, the electrical aspect of the nerve impulse, quite well. In fact, based on this understanding of the nerve impulse as an electrical phenomenon, it has been asserted that the electrical aspect of the nerve impulse is “the causal agent in [nerve impulse] propagation” (Hodgkin, 1964, p. 1148). However, this assertion is clearly the result of the assumption that the HH model represents the nerve impulse completely. Still, from the fact that the hypotheses that are studied (and thus the questions that are posed and answered about the nerve impulse) in electrophysiology are mainly electrical in nature, it does not follow that the nerve impulse is itself of an exclusively electrical nature. Experimental evidence has shown that the nerve impulse is not only manifested by an action potential, but also by mechanical and thermal changes, which could be of functional importance for nerve impulse initiation and/or propagation (Costa et al., 2018; Drukarch et al., 2018). Since the HH model cannot account for these non-electrical manifestations of the nerve impulse, it does not represent the nerve impulse completely. Therefore, concluding that the electrical action potential is the causal agent in nerve impulse propagation appears premature, since the fundamental cause(s) of this phenomenon might as well be of a non-electrical nature.

### The Engelbrecht Model: An Attempt at a General Unifying Model

The ample experimental evidence that the nerve impulse is accompanied by mechanical changes like axon swelling (e.g., Tasaki and Iwasa, 1982) and changes in intracellular pressure (Terakawa, 1985), and temperature changes (e.g., Howarth et al., 1968), has led to a resurgence of interest in the modeling of nerve impulse propagation in (neuro)scientific literature. Several new models have been developed that try to account for electrical, mechanical and/or thermal changes during nerve impulse propagation (Heimburg and Jackson, 2005; Rvachev, 2010; El Hady and Machta, 2015). Moreover, some (neuro)scientists have argued that all relevant aspects of the nerve impulse need to be incorporated, integrated and explained in a general model unifying different manifestations of the nerve impulse and their interaction(s) (Mueller and Tyler, 2014; Engelbrecht et al., 2018b). The idea behind the latter proposal, although not stated explicitly by the authors, seems to be that incorporating all relevant details about these manifestations and the processes underlying them enables the representation of the nerve impulse and its propagation in a complete and accurate way (something that could not be achieved by Hodgkin and Huxley (1952a) with their model). In this section, *as an illustration*, we will discuss a recently introduced general unifying model that is still in the process of development and refinement, the Engelbrecht model (Engelbrecht et al., 2016, 2018a,b). More specifically, we will answer the question whether this model can represent the nerve impulse and its propagation completely and accurately (without assuming that this is in fact the aim of the model).

As already mentioned above, the Engelbrecht model is neither the only model that attempts to model (non-)electrical manifestations accompanying the nerve impulse nor is it the first. However, the method for doing so distinguishes Engelbrecht and coworkers from other modelers like Heimburg and Jackson (2005); Rvachev (2010), and El Hady and Machta (2015). The latter modelers do not try to integrate existing models in a general unifying model in order to study (aspects of) nerve impulse propagation as Engelbrecht and coworkers do. This ‘non-integrating’ approach becomes clear in the article of El Hady and Machta (2015), who model the mechanical aspect of the nerve impulse as driven by the electrical aspect of this phenomenon, in the following quotes: “Our model does not assume a particular mechanism underlying the electrical component of the [action potential]” (p. 2) and “Our model does not require an underlying theory of how this electrical component arises. We emphasize that any traveling electrical wave will induce a co-propagating mechanical wave …” (p. 5)^2^. In this article, we conceptually analyze the attempt to integrate different models in order to obtain a general unifying model of nerve impulse propagation, since this accords with the intuition that neuroscience strives for a complete and accurate representation of complex neuroscientific phenomena. In the following, we will focus our discussion on the Engelbrecht model as an illustration of such an attempt.

**Figure 2:**
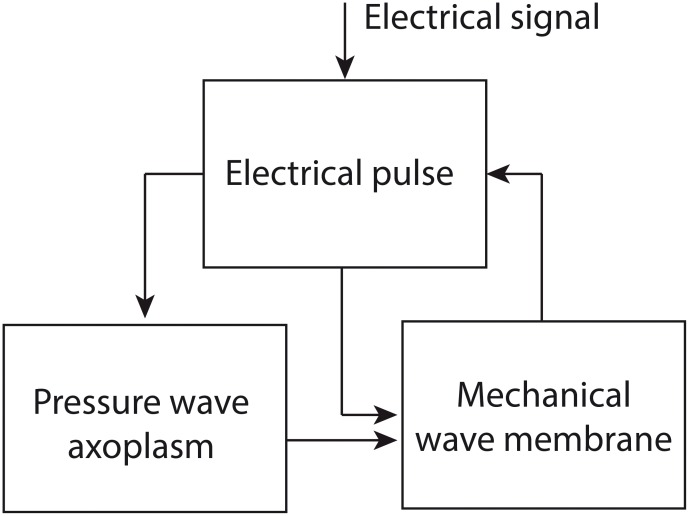
The proposed process of nerve impulse propagation in the Engelbrecht model. The input for the process is an electrical signal, which induces an electrical pulse. The electrical pulse generates a pressure wave in the axoplasmic fluid of the nerve fiber. The electrical pulse and the pressure wave together produce a mechanical wave in the neural membrane, which has a longitudinal and a transverse component. The mechanical wave can in turn have an influence on the electrical pulse. Figure adapted from Engelbrecht et al. (2018a).

In the Engelbrecht model, three waves are described mathematically: an electrical pulse, a pressure wave in the axoplasmic fluid of the nerve fiber and a mechanical wave in the neural membrane. The equations describing these waves are coupled via so-called coupling forces. The authors assume that the process of nerve impulse propagation proceeds as follows (Figure 2): an electrical signal above a certain threshold induces the generation of an electrical pulse, which in turn brings about a pressure wave in the axoplasm. The electrical pulse and the pressure wave together generate a mechanical wave in the neural membrane, which has a longitudinal and a transverse component. In its turn, the mechanical wave can influence the electrical pulse via mechanical activation; e.g., the opening of ion channels via mechanical input (Engelbrecht et al., 2016, 2018a,b).

Since the Engelbrecht model is a non-statistical mathematical model, it yields exact predictions that follow with certainty from the model’s starting assumptions. Thus, this model in which an ensemble of waves is described mathematically can be used to predict process characteristics of nerve impulse propagation (Engelbrecht et al., 2018b). However, the correctness of the predictions of such a model depends on the correctness of its assumptions. This means that even if the process characteristics that are predicted with the model are in agreement with experimental data, the value of these predictions remains relative to the following assumptions: (1) the assumption that the electrical signal triggers the described process of nerve impulse propagation, (2) the assumption that the distinct manifestations of the nerve impulse are the result of distinct processes, (3) the manifestations or processes that are assumed to be relevant for nerve impulse propagation, (4) the assumed order of the described processes, (5) the interactions between the described processes that are assumed to be relevant, and (6) the underlying assumptions about the way in which these processes interact. Although there is experimental evidence for the co-occurrence of intracellular pressure changes and mechanical displacements of the membrane during action potential propagation (Tasaki and Iwasa, 1982; Terakawa, 1985), the proposed models for the mechanisms underlying these co-occurring non-electrical waves are largely theoretical in nature (for a discussion of proposed models, see Drukarch et al., 2018). Thus, the value of the Engelbrecht model for predicting process characteristics of nerve impulse propagation depends on the correctness of assumptions that have not been experimentally tested as yet. Nevertheless, since we do not have experimental counterevidence against these assumptions or better evidence in favor of other assumptions, the choices of the modelers seem to make sense. However, these unverified assumptions have consequences for the conclusion whether the Engelbrecht model represents nerve impulse propagation accurately and completely: the agreement between the predictions of the model and experimental data does not imply a complete and accurate representation of this process as long as there is no better evidence for the assumptions made concerning completeness and accuracy (assumptions 1, 3, and 5 and assumptions 1, 2, 4, and 6, respectively). Thus, although the Engelbrecht model seems to include all relevant details about nerve impulse propagation, whether it can and does represent this complex process completely and accurately depends on the correctness of the assumptions about the initiation and the process of nerve impulse propagation.

In fact, it can be demonstrated that in its present form the Engelbrecht model does not represent the process of nerve impulse propagation accurately. To see why not, we need to zoom in on the components of the model. The model consists of existing mathematical models, which are used to describe the single processes involved in nerve impulse propagation (i.e., the electrical pulse, the axoplasmic pressure wave and the mechanical wave in the neural membrane, Figure 2). These models are integrated using coupling forces that are developed by the modelers themselves (Engelbrecht et al., 2016, 2018a,b).

For modeling the (propagating) electrical pulse, Engelbrecht and coworkers use the ‘FitzHugh-Nagumo’ model. This model is a simplification of the HH model. Instead of focusing on two ion currents (sodium and potassium), this model only describes one ion current. Both the FitzHugh-Nagumo model and the HH model can account for key characteristics of the action potential: the presence of a threshold for action potential generation, the all-or-none behavior of the action potential, etc. (Engelbrecht et al., 2016, 2018a,b). However, in both these models also an important assumption is made, namely that the membrane capacitance is constant (Hodgkin and Huxley, 1952a; FitzHugh, 1961). Since this assumption entails that the capacity current (first term in Equation 1) depends only on the membrane capacitance and the change in membrane voltage over time, the capacity current only plays a role when the membrane potential of the isolated nerve fiber is suddenly changed in voltage clamp experiments. When, after that, the membrane potential is kept constant, the first term in Equation 1 will become zero, and “the ionic current can be obtained directly from the experimental records” with the voltage clamp (Hodgkin et al., 1952, p. 426).

In the Engelbrecht model the (propagating) longitudinal component of the mechanical wave in the neural membrane is described using the model that Heimburg and Jackson (2005, 2006) developed. In order to explain the Heimburg-Jackson model, which is a thermodynamic model, some background information is needed. This model is based on the notion that under physiological conditions a membrane is predominantly in a fluid phase in which the lipids in the membrane are relatively disordered. Under these conditions, the membrane lipids are slightly above their melting temperature. A little below body temperature, the membrane lipids undergo a melting transition, and the fluid phase of the membrane transitions to a denser gel phase in which the lipids are more ordered. According to the Heimburg-Jackson model, the nerve impulse then is a localized electromechanical density pulse which consists of a traveling region of membrane in the gel phase in an environment of resting membrane in the fluid phase. During the density pulse, both the thickness and the area of the membrane change (compared to the resting membrane). These changes in membrane thickness and area lead to a change in the membrane capacitance during the nerve impulse (Heimburg and Jackson, 2005, 2006; Andersen et al., 2009; Appali et al., 2012; Wang et al., 2018). For a mathematical illustration of the dependence of the membrane capacitance on membrane thickness and area, see Equation 2.

**EQUATION 2. The membrane capacitance is a function of the membrane area and thickness.**

$$C_{m}\;=\;K_{m}\;*\;\varepsilon_{0}\;*\;\frac{A_{m}}{d_{m}}$$

In this equation, *C*_m_ is the membrane capacitance, *K*_m_ the dielectric constant of the membrane, *ε*_0_ the permittivity of free space, *A*_m_ the area of the membrane, and *d*_m_ the membrane thickness.

Although the assumption that the membrane capacitance is constant simplifies the study of the ionic current in voltage clamp experiments considerably and might be correct under the conditions in the voltage clamp, the result of this assumption is that the change in membrane capacitance during the nerve impulse is absent in the generally accepted explanation of the action potential (the electrical aspect of the nerve impulse) in terms of ionic currents through the membrane. However, in line with the Heimburg-Jackson model, the explanation of the action potential should at least be partly in terms of the changing membrane capacitance due to membrane area and thickness changes during the nerve impulse (“[s]ince the membrane is asymmetrically charged, these changes appear as a voltage pulse … and lead to a capacitive current” (Andersen et al., 2009, p. 107)) and not solely in terms of ions flowing across the membrane. Andersen et al. (2009, p. 105) phrase it even more firmly: “it seems that known changes in membrane area during the action potential are of an order of magnitude sufficient to account for the observed voltage changes during the action potential”.

Thus, two of the component models that are used in the Engelbrecht model, the Hodgkin-Huxley/FitzHugh-Nagumo model and the Heimburg-Jackson model, are incompatible due to inconsistencies with regard to the membrane capacitance, resulting in a logically inconsistent general unifying model. This implies that the model cannot be a fully accurate representation of reality, since such a representation should be free of inconsistencies. More specifically, in reality the capacitance of the neural membrane cannot be constant and change during the nerve impulse at the same time. Since the Engelbrecht model integrates incompatible models, it must represent the nerve impulse and its propagation inaccurately.

## Recommendations for (Future) Approaches to Studying Nerve Impulse Propagation

### Models as Tools to Study Nerve Impulse Propagation for Varying Purposes

Thus far we have seen that it is not straightforward to represent nerve impulse propagation accurately and completely using neuroscientific models. The HH model (in combination with information from subsequent studies on ion channels) does not represent the (propagating) nerve impulse completely, and whether it represents this phenomenon accurately is called into question in the Heimburg-Jackson model. Furthermore, we are not sure whether the Engelbrecht model, which unifies different manifestations of the nerve impulse and the interaction(s) between them, represents nerve impulse propagation completely. Moreover, since the Engelbrecht model attempts to integrate the incompatible HH model and Heimburg-Jackson model it does not represent this phenomenon accurately. Therefore, we want to present another perspective on the role of models in studying nerve impulse propagation by introducing two important aspects of models that have not entered the discussion yet: the (neuro)scientist that constructs and uses the model and the purpose for which the model is constructed and used.

If we look again at Figure 1, we see an electrical circuit. However, in and by itself this illustration does not represent the neural membrane. It is just an electrical circuit with some resistors, batteries and a capacitor. For it to become a model that represents the neural membrane, at least one scientist should intend to use this electrical circuit as such. Whether this model provides a useful representation of the neural membrane depends on the purpose for which the model is used by the scientist. For example, this model will not provide a useful representation of the neural membrane when it is used for the purpose of studying the molecular composition of the neural membrane, since an electrical circuit cannot provide information about this. On the other hand, the model of an electrical circuit does provide a useful representation of the neural membrane when it is used for studying the electrical manifestation of the nerve impulse as Hodgkin and Huxley (1952a) have shown in their work (see the section “The Hodgkin-Huxley Model”) and as is confirmed by many other scientists thereafter.

Philosopher of science Giere calls the conception of representation illustrated above the ‘intentional conception of scientific representation’, according to which: “Agents (1) intend; (2) to use model, M; (3) to represent a part of the world, W; (4) for some purpose, P” (Giere, 2010, p. 274). This formulation shows that a model cannot represent a phenomenon by itself. In addition, an agent (e.g., a scientist) is needed who uses the model as a representation of the phenomenon for a specific purpose s/he wants to achieve. More specifically, depending on the purpose for which an agent wants to use the model, s/he should specify which similarities are intended between the model and the phenomenon modeled, and how precise the model should correspond to experimental measurements of the phenomenon (Giere, 2004, 2006, 2010). Thus, a model does not represent reality accurately and completely simpliciter, but it represents it accurately and completely enough for a scientist to achieve a certain purpose. This approach to modeling implies that we do not have to incorporate as many details as possible in a model, but only those details that are relevant for reaching the goal of the model.

As already discussed in the section “The Hodgkin-Huxley Model”, the HH model does not provide an accurate representation of the action potential. It is also not meant to do so. The HH model is developed for the goal of describing the (propagating) action potential using a quantitative description of the membrane current. In the legend of Figure 1 and the explanation of Equation 1, we clearly see the similarities between the electrical circuit and the neural membrane that are specified by Hodgkin and Huxley (1952a). The details in this model are restricted to those that are important for achieving the goal of the model, i.e., not all the details about the structure and function of the neural membrane are included in the model, only those that are important for describing the electrical aspect of the nerve impulse. The data that were used for the development of the HH model, Equation 1^3^, were obtained in voltage clamp experiments (in which the voltage is kept constant). Using the resulting equation, the curve for the (propagated) action potential (i.e., the voltage change that cannot be measured under a voltage clamp) can be calculated. The curve that is produced using Equation 1 corresponds well with the (propagated) action potential measured in isolated nerve fibers, providing evidence that the action potential can indeed be described using the HH model (Hodgkin and Huxley, 1952a). However, since Hodgkin and Huxley chose to develop theoretical equations for the sodium and potassium conductance of the neural membrane which they fitted to experimental data (by lack of sufficient knowledge about the membrane), they could only speculate about the mechanism of permeability that is responsible for these changes (Hodgkin and Huxley, 1952a). Thus, Hodgkin and Huxley could not use their model for explaining the molecular mechanism underlying the permeability changes of the neural membrane during the nerve impulse. They could not specify the similarities between the (conductances/resistors of the) electrical circuit and the neural membrane accurately and/or completely enough for this purpose.

The nerve impulse can also be modeled for quite different purposes. Heimburg and Jackson, for instance, aim to develop a thermodynamic model of nerve impulse propagation (Andersen et al., 2009; Appali et al., 2012). Their model is presented in the section “The Engelbrecht Model: An Attempt at a General Unifying Model” as a model of the longitudinal component of the mechanical wave in the neural membrane, like it is used in the Engelbrecht model. However, as the goal of developing a thermodynamic model indicates and as already became clear during the discussion in the section “The Engelbrecht Model: An Attempt at a General Unifying Model”, the Heimburg-Jackson model encompasses more than only the description of a mechanical wave. Rather, it aims to provide a comprehensive description of the propagating nerve impulse in terms of mechanical and electrical changes (and other changes, like thermal ones). According to this thermodynamic model, the nerve impulse is “a self-sustaining and localized density pulse with a moving segment of the nerve membrane in the gel [phase]” or ‘soliton’ which can propagate without loss of energy through the neural membrane (Andersen et al., 2009, p. 107). This soliton is associated with a heat release when the membrane transitions from the fluid to the gel phase and a subsequent heat reabsorption when the membrane transitions back to the fluid phase. Since no net heat is gained from or lost to the environment during soliton propagation, the soliton is classified as an adiabatic pulse (since, in thermodynamics, an adiabatic process has precisely these characteristics). Electrical, mechanical and thermal changes are all macroscopic features of a soliton in the neural membrane, which can be measured during its propagation and should be in agreement with the characteristics of an adiabatic process (Heimburg and Jackson, 2005, 2006; Andersen et al., 2009; Appali et al., 2012).

Since the Heimburg-Jackson model is a thermodynamic model, with this model “a macroscopic description” can be given of the nerve impulse in the neural membrane “in terms of [macroscopic] quantities that are detectable directly by our senses and instruments”, like temperature and volume (Giancoli, 2009, p. 454). Thus, this model is not aimed at identifying the microscopic constituents involved in the process of nerve impulse propagation that are responsible for the macroscopic quantities that are experimentally measured. Instead, its goal is to describe nerve impulse propagation macroscopically in terms of these experimental measures based on thermodynamic laws. More specifically, this macroscopic description should meet the thermodynamic laws applied to an adiabatic process. Every macroscopic measurement of the propagating nerve impulse (e.g., electrical, mechanical, thermal, etc.) provides a test of the correctness of approaching the nerve impulse as an adiabatic pulse. However, whether nerve impulse propagation is an adiabatic process still needs to be proven. In particular, it has to be experimentally demonstrated that the heat production and subsequent reabsorption during propagation is exactly reversible, which is difficult due to technical limitations of the available experimental instruments (Andersen et al., 2009; Drukarch et al., 2018).

According to this approach to models, in which the purpose for which a model is developed and used is taken into account, the HH model cannot be considered superior, equivalent or inferior to the Heimburg-Jackson model. Since models should be judged based on whether they can achieve certain goals, and since these two models are not developed and used for the same goal, it does not make sense to assess them comparatively. However, this is not a problem for the progress of (neuro)science. For this, it is first and foremost important to learn lessons from these models and use insights obtained with them, e.g., for understanding the nerve impulse better, developing new models or designing new experiments.

Going back to the Engebrecht model, the general unifying model that attempts to integrate the HH model and the Heimburg-Jackson model, the question that still needs to be answered is: for which purpose is this model developed? According to Engelbrecht et al. (2018b, p. 32): “[i]n terms of complexity, the goal is to formulate a model that will be able to describe an ensemble of waves of different physical origin (electrical and mechanical)”. If the aim of the model is interpreted in terms of coupling the phenomenological, mathematical descriptions of the measured curves of the electrical pulse and the mechanical waves independently of their physical basis, this goal can be achieved. But if these phenomenological, mathematical descriptions are interpreted in terms of their physical basis, problems arise. In the Engelbrecht model it is assumed that the different aspects of the nerve impulse are the result of distinct processes. The single processes are described in distinct component models unified in the general model. However, one of these component models, the Heimburg-Jackson model, does not describe a purely mechanical wave in the neural membrane, as is suggested in the Engelbrecht model: in fact, its description also encompasses the electrical pulse. Moreover, the Heimburg-Jackson model is not compatible with the HH model with regard to the physical basis of the electrical pulse. Of note, the Heimburg-Jackson model suggests that the different manifestations of the nerve impulse could be features of a single process instead of being the result of distinct processes, as is assumed in the Engelbrecht model (although this thermodynamic model itself does not provide insights in the molecular basis of the process of nerve impulse propagation). At the moment this remains to be elucidated experimentally. Due to these problems, the equations in the Engelbrecht model cannot be interpreted in terms of their physical basis.

Still, using the ensemble of phenomenologically described waves, the Engelbrecht model can provide insights in the mathematically *possible* process characteristics and, more specifically, interactions between the single waves based on (future) experimental observations of the spatiotemporal relations (or hypothetical spatiotemporal relations) between the electrical and mechanical manifestations of the nerve impulse (Engelbrecht et al., 2018a). These insights could in turn be used to guide future investigations in order to identify the *actual* interactions between the waves and the microscopic constituents that are responsible for them.

### The Construction of a Comprehensive Framework of Nerve Impulse Propagation

In the section “The Engelbrecht Model: An Attempt at a General Unifying Model”, we have shown that the Engelbrecht model cannot provide a consistent picture of the nerve impulse and its propagation, since it integrates component models that are incompatible due to an inconsistency regarding the membrane capacitance. Indeed, in general, if a general unifying model is built using component models of single aspects of the nerve impulse, it will be virtually impossible to construct a model that provides a consistent picture of nerve impulse propagation. The reason for this is the fact that the component models are developed for varying purposes which require different and often conflicting idealizing assumptions in order to achieve those purposes^4^. This suggests that a comprehensive framework of nerve impulse propagation will not be accomplished by developing a single general unifying model. An alternative approach may therefore be required to develop a comprehensive framework of nerve impulse propagation. Here, we suggest one that is based on an account of philosopher of science Hochstein (2016), who argues that a neuroscientific mechanism (e.g., nerve impulse propagation) cannot be mechanistically explained^5^ in one model but, instead, that many (sometimes contradictory) models are needed to provide such an explanation.

Understanding a complex phenomenon like nerve impulse propagation is not easy. Models need to be developed in order to make parts of this phenomenon comprehensible to us. To achieve this, idealizing assumptions need to be made in accordance with the purpose for which the models are developed. With these models, that provide partial explanations of nerve impulse propagation, a comprehensive framework can be built in the way a mosaic is constructed using several tiles^6^. Thus, in the resulting framework the overall explanation of nerve impulse propagation can be inferred from a set of models like the picture represented by a mosaic can be inferred from a collection of tiles. All models in the framework put constraints on the others. More precisely formulated, the parts of the models that successfully represent a part of nerve impulse propagation put constraints on the other models within the collection. However, the resulting comprehensive framework will not look like a puzzle of which the pieces fit perfectly together. Instead, (at least at the start) the framework will have gaps due to the fact that not all aspects of the nerve impulse are or can be modeled (yet). In addition, some of the models within the framework will overlap or will be based on conflicting assumptions. As a result, the explanation of nerve impulse propagation needs to be inferred from the piecemeal and sometimes contradictory representation of this phenomenon in the distinct models that constitute the comprehensive framework. Moreover, the comprehensive framework will evolve over time, and the explanation of nerve impulse propagation can change due to the addition of the latest (neuro)scientific insights to the framework or the removal of erroneous models. In addition, the explanation that is given of nerve impulse propagation using a comprehensive framework will also depend on the purpose for which the explanation is employed, as discussed in the section “Models as Tools to Study Nerve Impulse Propagation for Varying Purposes”.

The suggested (construction of a) comprehensive ‘mosaic’ framework of nerve impulse propagation might seem very unsatisfying compared to the ideal of a single, logically consistent general unifying model. However, our current explanation of the action potential, the electrical aspect of the nerve impulse, is already the result of a framework consisting of a set of distinct models that has developed over time. In order to illustrate that the above suggested approach to develop comprehensive frameworks for explaining complex phenomena may work in neuroscientific practice, and to show how such a comprehensive framework is built, we will sketch the history of the discovery and subsequent study of the sodium channel after the introduction of the HH model and discuss this history in the context of the construction of a comprehensive framework of the action potential. The following discussion is based on reviews by Barchi (1988) and Trumpler (1997).

With the HH model the sodium conductance of the neural membrane could be modeled. However, in the time period in which this model was introduced, the physical basis underlying the sodium conductance was unknown, leaving a gap in the explanation of the action potential. It took decades before this sodium conductance could be studied in more detail using the patch clamp technique, with which sodium currents through small patches of the membrane can be measured (we already discussed this briefly in the section “The Hodgkin-Huxley Model”). However, before it could be concluded that the patch clamp measurements were related to the representation of the action potential in the HH model, the relation between the ‘macroscopic’ currents measured with the voltage clamp and the ‘microscopic’ currents measured with a patch clamp had to be established. Assuming that the microscopic currents measured in patch clamp experiments are the result of identical sodium channels that function independently, the average of the sum of many microscopic current measurements should be in accordance with the characteristics of a macroscopic sodium current measured with a voltage clamp. This was shown to be the case, thereby illustrating that the microscopic sodium conductance is responsible for the macroscopic one (Sigworth and Neher, 1980). Here, we see clearly that the characteristics of the macroscopic sodium current were used as a constraint for the microscopic sodium currents in order to determine whether the results obtained with the patch clamp could be added to the framework that explains the action potential.

However, the framework explaining the action potential did not only consist of an electrophysiological representation of sodium conductance based on electrophysiological data and models. In addition, models representing the molecular structure of the sodium channel, which was assumed to be responsible for the sodium conductance, were developed. One of the first things known about the purified sodium channel protein, which could be identified using neurotoxins like radiolabeled tetrodotoxin, was its molecular weight. The structure of this tetrodotoxin-binding protein was not established at that time, but using patch clamp recordings it was shown that the protein has biophysical properties that correspond to those expected for the “physiologically defined [sodium] channel” (Rosenberg et al., 1984, p. 5597), demonstrating that this protein fits in the framework that explains the action potential. Later, the genetic code of the protein was identified, the amino acids that correspond to this genetic code were determined, and models of the protein structure were developed (Noda et al., 1984; Guy and Seetharamulu, 1986). Thus, the molecular structure of the sodium channel was represented in models that do not represent sodium currents across the membrane. Moreover, although the models of the protein structure were based on the experimental evidence about the genetic code and the corresponding amino acids, these models were only partially overlapping (and thus at some points contradictory) due to different considerations of the scientists. Since both models were in agreement with the available experimental data, both can be considered part of the framework explaining the action potential at that time. Such models of the sodium channel structure were in turn used to suggest which structural parts of the sodium channel are involved in channel activation and inactivation (Noda et al., 1984; Guy and Seetharamulu, 1986; Stühmer et al., 1989). These suggestions could be tested experimentally by changing the molecular structure of the sodium channel (using genetic engineering) and investigating its resulting electrophysiological characteristics (using voltage- and patch clamp recording) (Stühmer et al., 1989). By exploring relationships between the molecular structure and electrophysiological characteristics of the sodium channel in this way, the framework explaining the action potential could be complemented with new pieces of experimental information about (the kinetics of) sodium channel gating. This information could then be used to limit the models of the sodium channel structure in the framework to those that are in agreement with the latest experimental data (but which still could be contradictory at other points).

Thus, the history starting with a sodium conductance in the HH model and resulting in the discovery and study of the molecular structure of the sodium channel shows how a comprehensive framework has been built from distinct models that inform and constrain each other. This set of models can be used to explain the action potential without integrating all these models into one general unifying model. Of course, the explanation of the action potential is not only based on models that represent the molecular structure or electrophysiological characteristics of sodium channels, but this example suffices to illustrate that the overall explanation is inferred from distinct models that each provide part of the explanation. In a comparable way a comprehensive framework of nerve impulse propagation can be constructed, which may or may not include the HH model, the Heimburg-Jackson model and the Engelbrecht model.

## Conclusion

From a critical examination of the Engelbrecht model we have drawn two conclusions, and combining these conclusions with recent insights from philosophy of science, we have made two recommendations for the study of nerve impulse propagation. The first conclusion of our analysis is that attempts to develop models that represent nerve impulse propagation accurately and completely appear unfeasible. Instead, models are and should be used as *tools* to study nerve impulse propagation for selected goals, representing the nerve impulse accurately and completely enough to achieve these goals. The second conclusion is that since models of distinct aspects of the nerve impulse, developed for selected purposes, require different and often incompatible idealizations, they cannot be integrated in a general unifying model that consistently models nerve impulse propagation in all its details. Instead of unifying such models in one general model, we suggest that a comprehensive ‘mosaic’ framework of nerve impulse propagation should be constructed using distinct models. From this collection of models the explanation of this complex phenomenon can be inferred based on the piecemeal and sometimes contradictory representation of it in the distinct models. This explanation of nerve impulse propagation can change over time due to the addition of models to, or the removal of models from, the comprehensive framework.

However, although a general unifying model cannot provide an all-encompassing explanation and representation of nerve impulse propagation, this does not mean that it cannot fulfill a function *in* a comprehensive framework of nerve impulse propagation. It can be of additional value in the framework if it serves a purpose that other models cannot. For instance, a general unifying model may provide insight in the *causal relations* between the different aspects of the nerve impulse, which is something that models of single aspects of the nerve impulse cannot capture. In such a general unifying model not all details regarding nerve impulse propagation need to be incorporated, but instead the incorporation of details should be limited to the ones that are relevant to the study of causal relations. However, as follows from the discussion here, there are some requirements that should be met before a general unifying model can be of value for the study of causal relations. The first requirement is that the different manifestations of the nerve impulse are actually results of separate processes and not just distinct features of a single process, since, in the latter case, a model focusing on this process can already capture the causal relations. This should be sorted out experimentally, which is not straightforward to do in the case of the nerve impulse, since it is currently not possible to study the electrical and mechanical aspects of the nerve impulse in isolation in nerves using experimental interventions. If the different manifestations of the nerve impulse turn out to be the result of distinct processes, the second requirement is that the models of the separate processes that are unified in the general unifying model should offer compatible perspectives on the causal relations that are studied, since these causal relations cannot be clarified if they are described in a logically inconsistent way in the general unifying model.

Thus, the motivation, given in the “Introduction”, to develop a general unifying model in order to obtain insights in nerve impulse propagation that cannot be acquired with models that focus only on one or a few aspects of the nerve impulse without studying the interactions between these aspects, still stands. However, in this article, we have shown that these insights are not achieved by incorporating as many details as possible about nerve impulse propagation, but by focusing on the goals that cannot be reached by compartmentalized models and by incorporating details accordingly. The Engelbrecht model provides a good example here. With this model, insights in the mathematically possible interactions between the electrical and mechanical manifestations of the nerve impulse can be provided based on spatiotemporal relations between them, which requires only the phenomenological and mathematical description of these manifestations. However, since these phenomenological descriptions cannot be interpreted in terms of their physical basis, this model cannot provide an accurate and complete representation of nerve impulse propagation.

## Author Contributions

LH wrote the article. LH, HR, and BD discussed several versions of the article. In these discussions, HR and BD provided feedback and made additional contributions, both contributed equally to the article. All authors approved the manuscript for publication.

## Conflict of Interest Statement

The authors declare that the research was conducted in the absence of any commercial or financial relationships that could be construed as a potential conflict of interest.

## Footnotes

1 In the section “The Engelbrecht Model: An Attempt at a General Unifying Model” it will turn out, however, that not every (neuro)scientist agrees with this conclusion.

2 Of course, El Hady and Machta (2015) have ideas about the mechanism underlying the electrical aspect of the nerve impulse. More specifically, they propose two possibilities (p. 2): “Our model does not assume a particular mechanism underlying the electrical component of the [action potential]. Indeed, we expect that the surface waves we predict would accompany the [action potential] predicted by Hodgkin and Huxley and the cable theory, even if they do not contribute to neuronal function. However, our results also allow for the possibility that the mechanical changes that accompany these surface waves feed back and influence the electrical [action potential], giving them functional importance”.

3 Note that Equation 1 is a simplification of the original equation developed in the HH model. Moreover, for modeling a propagating action potential Equation 1 has to be extended. These points are also discussed in the section “The Hodgkin-Huxley Model”.

4 This is one of the problems with regard to the neuroscientific explanation of a mechanism using a single model which is discussed by Hochstein (2016). Craver and Kaplan (2018) discuss this problem in the context of developing norms of completeness for mechanistic explanations.

5 “[A] model provides [a mechanistic] explanation when it identifies four essential features of the mechanistic system [e.g., nerve impulse propagation]: (1) The parts of the system. (2) The way in which these parts are spatially and temporally organized within the system. (3) The operations that go on between the relevant component parts. (4) The resulting phenomenon produced by the system.” (Hochstein, 2016, p. 1393).

6 Craver (2007) introduces the metaphor of a mosaic in the context of presenting a model of the unity of neuroscience (which is aimed to reflect neuroscientific practice).
